# Impact of Large Language Models on Medical Education and Teaching Adaptations

**DOI:** 10.2196/55933

**Published:** 2024-07-25

**Authors:** Li Zhui, Nina Yhap, Liu Liping, Wang Zhengjie, Xiong Zhonghao, Yuan Xiaoshu, Cui Hong, Liu Xuexiu, Ren Wei

**Affiliations:** 1 Department of Vascular Surgery, The First Affiliated Hospital of Chongqing Medical University, Chongqing, China; 2 Department of General Surgery, Queen Elizabeth Hospital, St Michael, Barbados; 3 Department of Ultrasound, The First Affiliated Hospital of Chongqing Medical University, Chongqing, China; 4 Department of Nuclear Medicine, The First Affiliated Hospital of Chongqing Medical University, Chongqing, China; 5 Department of Acupuncture and Moxibustion, Chongqing Traditional Chinese Medicine Hospital, Chongqing, China; 6 Department of Anesthesia, The First Affiliated Hospital of Chongqing Medical University, Chongqing, China; 7 Department of Neonatology, Children’s Hospital of Chongqing Medical University, Chongqing, China

**Keywords:** large language models, medical education, opportunities, challenges, critical thinking, educator

## Abstract

This viewpoint article explores the transformative role of large language models (LLMs) in the field of medical education, highlighting their potential to enhance teaching quality, promote personalized learning paths, strengthen clinical skills training, optimize teaching assessment processes, boost the efficiency of medical research, and support continuing medical education. However, the use of LLMs entails certain challenges, such as questions regarding the accuracy of information, the risk of overreliance on technology, a lack of emotional recognition capabilities, and concerns related to ethics, privacy, and data security. This article emphasizes that to maximize the potential of LLMs and overcome these challenges, educators must exhibit leadership in medical education, adjust their teaching strategies flexibly, cultivate students’ critical thinking, and emphasize the importance of practical experience, thus ensuring that students can use LLMs correctly and effectively. By adopting such a comprehensive and balanced approach, educators can train health care professionals who are proficient in the use of advanced technologies and who exhibit solid professional ethics and practical skills, thus laying a strong foundation for these professionals to overcome future challenges in the health care sector.

## Introduction

Technological advancements have significantly shaped medical education, leading to transformative shifts in approaches to teaching and learning. The recent rapid evolution of artificial intelligence (AI) technology has entailed unprecedented changes and challenges in this field, particularly due to the emergence of large language models (LLMs). LLMs, which are extensively trained on vast text data sets, are advanced AI systems best exemplified by OpenAI’s GPT series, Google’s Gemini, and Twitter’s Grok [1]. By using complex neural network architectures, especially those based on transformer design, LLMs can identify and replicate subtle linguistic nuances. Their capabilities extend beyond the generation of text; they can also produce corresponding images or even videos based on text inputs, thereby addressing a wide variety of linguistic tasks. Notably, the use of LLMs in medical education is increasingly becoming a focal point of global research and discussion [2,3].

In the context of medical education, LLMs can answer questions regarding medical concepts, generate simulated cases, assist researchers, and support clinical decision-making [4,5]. These applications not only optimize the efficiency of teaching but also offer students a more personalized, digital, and adaptable learning experience [6]. However, as this tool is integrated into medical education, challenges pertaining to the accuracy of information, privacy, potential overreliance on such technology, and academic integrity have emerged [7].

This viewpoint article, which is based on current international research, thoroughly explores the impacts associated with the integration of LLMs into medical education. The article also analyzes in depth the adaptive changes that teaching methods should make in response to these opportunities and challenges. The primary goal of this article is to provide a nuanced perspective on the use of technological innovation in medical education and to serve as a reference for future teaching practices and strategies.

## Opportunities in Medical Education

**Figure 1.**
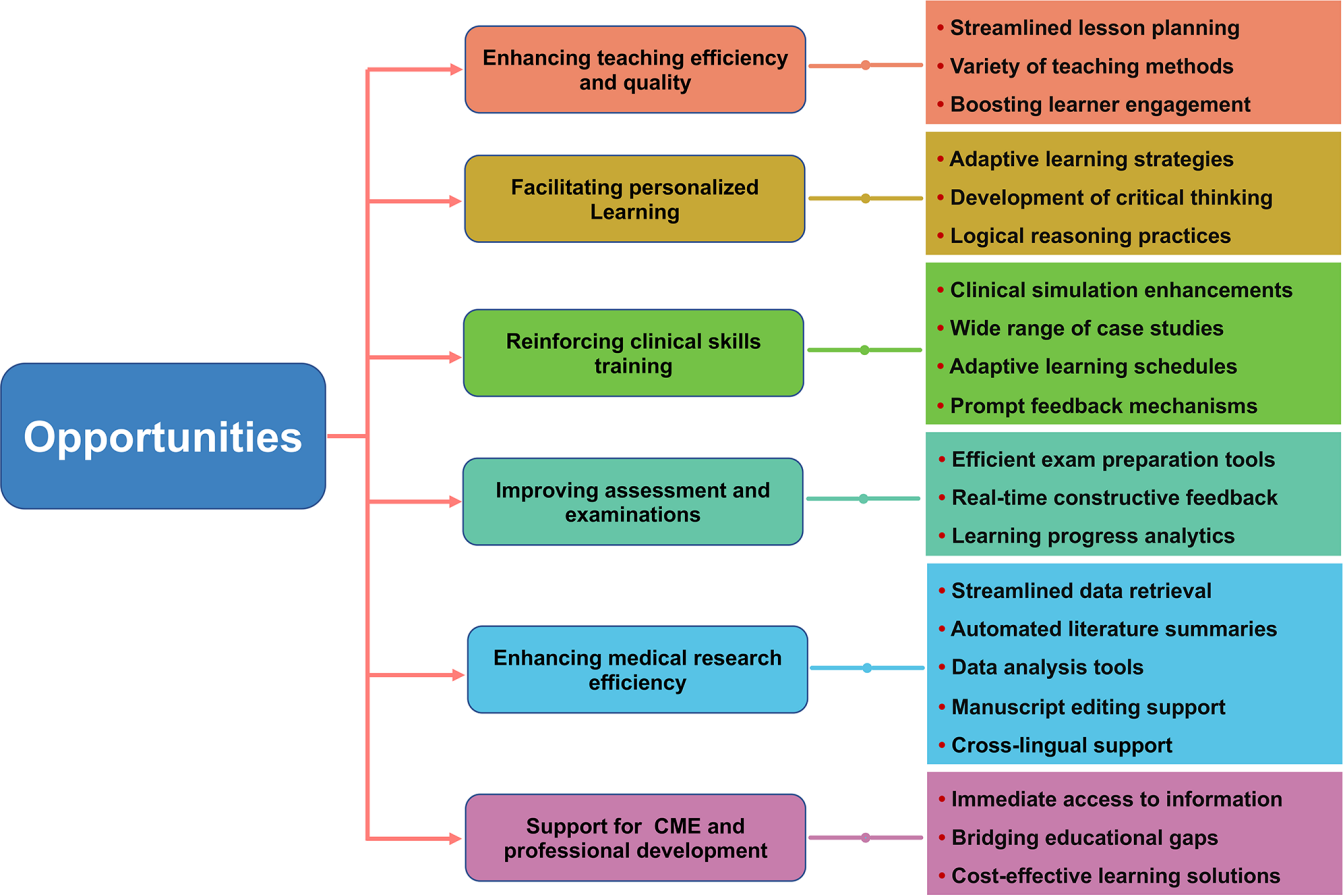
Opportunities associated with the use of large language models in medical education. CME: continuing medical education.

The various uses of LLMs are gradually changing traditional medical education. The opportunities presented by LLMs in the context of medical education are illustrated in Figure 1.

## Enhancing the Efficiency and Quality of Teaching

The integration of LLMs into medical education heralds a transformative advance in the efficiency and quality of instruction. A study that focused on the ChatGPT 3.5 platform in the context of a problem-based learning teaching model revealed that the application of ChatGPT significantly enhanced collaboration and participation among members of learning groups. This approach also increased students’ motivation, encouraging them to pose questions more actively [8]. A randomized controlled study that compared ChatGPT-assisted learning with traditional literature-based learning reported that students’ examination scores significantly improved as a result of ChatGPT-assisted learning [9]. By leveraging these advanced tools, educators can swiftly craft comprehensive syllabi, develop engaging lecture materials, and create detailed textbooks. This approach is particularly effective within the frameworks of case-based learning and problem-based learning, which emphasize contextual and practical learning [10,11]. LLMs offer 2 advantages in educational settings. First, they can generate visual content, such as illustrative diagrams to depict disease progression or 3D models of anatomical structures, and they can even source existing educational imagery on the internet to meet specific learning objectives. This capability not only enriches teaching materials but also caters to diverse learning styles, thus facilitating students’ retention and understanding of complex concepts. Second, LLMs excel in the tasks of analyzing and interpreting visual data. They can help educators and students decipher complex medical images such as computed tomography (CT) or magnetic resonance imaging (MRI) images. Through such digital and visually driven learning experiences, LLMs significantly enhance students’ comprehension and retention of information regarding challenging medical subjects, such as pathology or clinical diagnosis. The use of LLMs in medical education substantially improves the efficiency of preparing instructional material as well as the quality of the material itself. By enhancing students’ collaboration skills and learning motivation, this approach effectively improves learning outcomes.

## Facilitating Personalized Learning

Personalized learning involves tailoring instructional content and strategies to the specific needs, abilities, interests, and learning styles of students. Compared to traditional educational models, LLMs offer a more flexible, digital, and immediate approach to learning [12]. Traditional education often relies on standardized curriculum structures and educator guidance, while LLMs can adapt to each student’s unique learning needs and style, thereby providing students with customized guidance and feedback. Such assistance not only enhances student engagement but also helps students grasp knowledge at their own pace [13,14].

Xu et al [15], who conducted interviews with 6 higher education professors and 3experts in information and communication technology, revealed that ChatGPT significantly improves students’ cognitive skills, such as their ability to process information, solve problems, and understand concepts. It also enhances students’ noncognitive skills, such as motivation and self-efficacy, and facilitates their development of metacognitive skills, thus helping students plan and adjust their learning. These improvements are crucial for enhancing personalized and interdisciplinary education in higher education [15]. This support is particularly crucial for students in dynamic fields such as medicine, in which context triggering intrinsic motivation for self-learning and offering a timely understanding of the latest research findings in depth are essential.

Moreover, ChatGPT facilitates a discussion-based learning approach that is closely connected to real-world scenarios. This type of interaction not only facilitates knowledge acquisition but also serves as a platform through which students can engage LLMs as debate partners, thereby fostering the development of critical thinking skills by enabling students to present and evaluate different viewpoints [16]. Accordingly, students can use LLMs to create a tailored learning journey based on their own interests, knowledge gaps, and learning pace, thereby crafting a personalized educational path that aligns with their individual needs. Additionally, a study that evaluated students’ reasoning abilities by referencing published case reports and simulated clinical cases revealed that ChatGPT significantly enhances the clinical reasoning abilities of medical professionals [17]. This kind of reasoning ability is developed through personalized feedback based on the individual learning progress and needs of each person. Through these digital and customized teaching solutions, LLMs effectively meet the personalized learning needs of students. This approach can enhance students’ engagement and promote the development of crucial skills such as critical thinking and logical reasoning, thereby helping students overcome the challenges entailed by an ever-evolving knowledge landscape effectively.

## Reinforcing Clinical Skills

Traditional clinical skills training relies heavily on real clinical cases or standardized patients (SPs), which can allow students to interact with patients and address authentic clinical challenges directly. However, this approach has significant drawbacks, including high costs, temporal and spatial constraints, the inclusion of only a limited variety of medical conditions, issues related to patient privacy, and the inability to provide timely feedback regarding learning outcomes.

The integration of LLMs into clinical skills training can effectively address these limitations, as demonstrated by a pioneering study conducted in China. The use of ChatGPT to simulate SPs not only addresses the scarcity of SPs but also obviates the need for supplementary training, thereby conserving substantial human and material resources. Crucially, LLMs such as ChatGPT can emulate a diverse array of SPs, thereby delivering intelligent, articulate, and vivid responses that are tailored to specific scenarios [18]. This innovation enables students to engage in repetitive practice in a low-risk environment that can obviate concerns regarding patient welfare and privacy, thereby enhancing students’ diagnostic reasoning and communication skills [19].

The use of LLMs to simulate clinical patients offers other significant advantages. First, given that the accessibility of LLMs—for instance, ChatGPT 3.5 and Microsoft Gemini—do not require registration and can be seamlessly integrated into browsers—clinical simulation exercises can become ubiquitous, thus transcending the traditional confines of clinical settings. This flexibility significantly enhances the adaptability of learning [12]. Second, the ability of LLMs to replicate a spectrum of diseases, ranging from common ailments to rare conditions, broadens the scope of educational content and experiences, thereby addressing students’ lack of exposure to rare clinical cases. According to Scherr et al [5], LLM simulations facilitate the early development of independent diagnostic and therapeutic reasoning among medical students. Furthermore, LLMs can adaptively refine their responses based on student interactions, thereby offering a more authentic reflection of real-world patient-provider communication than is possible using conventional, static simulation models. Moreover, LLMs provide an inexhaustible supply of free simulation opportunities, thus democratizing access to medical education, particularly for students from economically disadvantaged backgrounds or for institutions with limited resources [5].

Although LLMs currently do not extend to procedural skill training in medical education, such as training in surgical tasks, including debridement and suturing, the prospective integration of LLMs with virtual reality and augmented reality technologies could represent a transformative advance in this regard. This combination could facilitate the establishment of a multidimensional, digital, and highly authentic simulation learning environment, thereby leading to the advent of a new era of innovative teaching methodologies and content in the context of medical education. Such advancements can address students’ diverse educational needs, enrich the learning landscape, and imbue the domain with fresh momentum.

## Improving Medical Teaching Assessments and Examinations

Traditional medical education assessments, which include standardized examinations, interviews, and clinical skills evaluations, require considerable preparation, detailed grading, and extensive time to compile students’ scores. In contrast, LLMs such as ChatGPT represent a dynamic shift in this context by streamlining the creation of diverse, integrated assessment questions; they are, thus, closely aligned with instructors’ needs. A study conducted in Hong Kong, Singapore, and the United Kingdom highlighted the ability of the ChatGPT to generate 50 multiple-choice questions rapidly, with the questions thus generated exhibiting a quality comparable to that of questions developed by university professors while offering a significant reduction in preparation time [20]. This efficiency extends to case-based questions, in which context the outputs of ChatGPT have been reported to be equally robust [21].

The evolving research on LLMs has focused on their precision regarding addressing a spectrum of medical questions, thus highlighting their ability to enhance educational assessments. The success of the ChatGPT in passing rigorous examinations regarding medical theory, such as the United States Medical Licensing Examination and the Objective Structured Clinical Examination in Singapore, highlights the proficiency of this LLM in domain-specific knowledge and its potential applicability across a variety of medical assessments [22,23]. In addition to generating questions, LLMs excel in the tasks of delivering immediate, detailed feedback, identifying student errors, and highlighting areas for improvement [24]. Furthermore, LLMs provide deep insights into examination results, thereby offering educators real-time insights into the learning challenges faced by students. This analytical capability fosters a more nuanced understanding of student performance, thus enabling educators to tailor their teaching strategies effectively [25].

The integration of LLMs into medical education heralds a more efficient, personalized assessment process, thereby decreasing educators’ workloads while providing students with a clearer comprehension of their academic progress. This innovative approach enriches the educational experience, shifting from a conventional focus on scores to a more holistic perspective on the efficacy of learning and teaching.

## Enhancing the Efficiency of Medical Research

Information retrieval in the context of medical research has long been associated with challenges such as information overload, time-consuming classification processes, and a lack of personalized suggestions. Due to their intelligent information retrieval capabilities, LLMs can offer more precise and personalized literature search suggestions based on researchers’ queries. This technology can help researchers swiftly locate relevant information and effectively mitigate issues pertaining to information overload [26]. Moreover, LLMs can summarize lengthy papers and offer reasonable advice regarding research methods, experimental design, and statistical analysis, thereby accelerating the research process and enhancing the efficiency of research [27]. Additionally, LLMs can facilitate data analysis and visualization, enabling researchers to interpret and summarize research results efficiently and streamlining the research workflow [2].

LLMs also provide cross-lingual assistance to researchers from non-English-speaking countries, thereby improving the quality of research [28-30]. Numerous studies have indicated that ChatGPT can help researchers refine papers and that the ability of LLMs to write abstracts is comparable to that of humans, thus highlighting the potential of this technology regarding providing support for academic writing [31-33]. Indeed, scholar King [34] successfully published a paper produced with ChatGPT that required no further language editing. Additionally, ChatGPT can help researchers draft cover letters, thus enabling them to convey the significance and relevance of their research effectively when submitting papers to journals [35,36]. The combined impact of these capabilities can significantly boost the ability of researchers from non-English-speaking backgrounds to engage in international academic communication and support the results of their research.

## Support for Continuing Medical Education and Professional Development

Rapid advancements in the field of medicine require health care professionals to engage in lifelong learning to enhance their professional competence continuously. In this context, LLMs represent an efficient, convenient, and flexible approach to continuing medical education. For instance, LLMs can systematically and specifically help medical professionals remain up-to-date regarding the most recent developments in medical research, treatment guidelines, and clinical practices [24]. Several comparative studies focusing on various LLMs and search engines have consistently revealed that LLMs can generate evidence-based medical recommendations tailored to specific topics [37,38]. The experience of clinical doctors is often limited due to their frequent encounters with common and prevalent cases, which often causes them to lack sufficient expertise regarding rare diseases or emerging epidemics, as was observed at the onset of the COVID-19 pandemic. In this regard, LLMs can address the knowledge gaps of health care professionals by collecting global medical information or providing clinical experiences with respect to the diagnosis and treatment of specific diseases. By using LLMs, health care professionals can pursue continuing education in a manner that requires minimal economic and time investments, thereby enhancing their clinical decision-making capabilities. This approach ensures that professionals in the field of health care can effortlessly remain up-to-date in terms of their knowledge and skills despite their demanding clinical schedules [39].

## Challenges Associated With Medical Education

**Figure 2.**
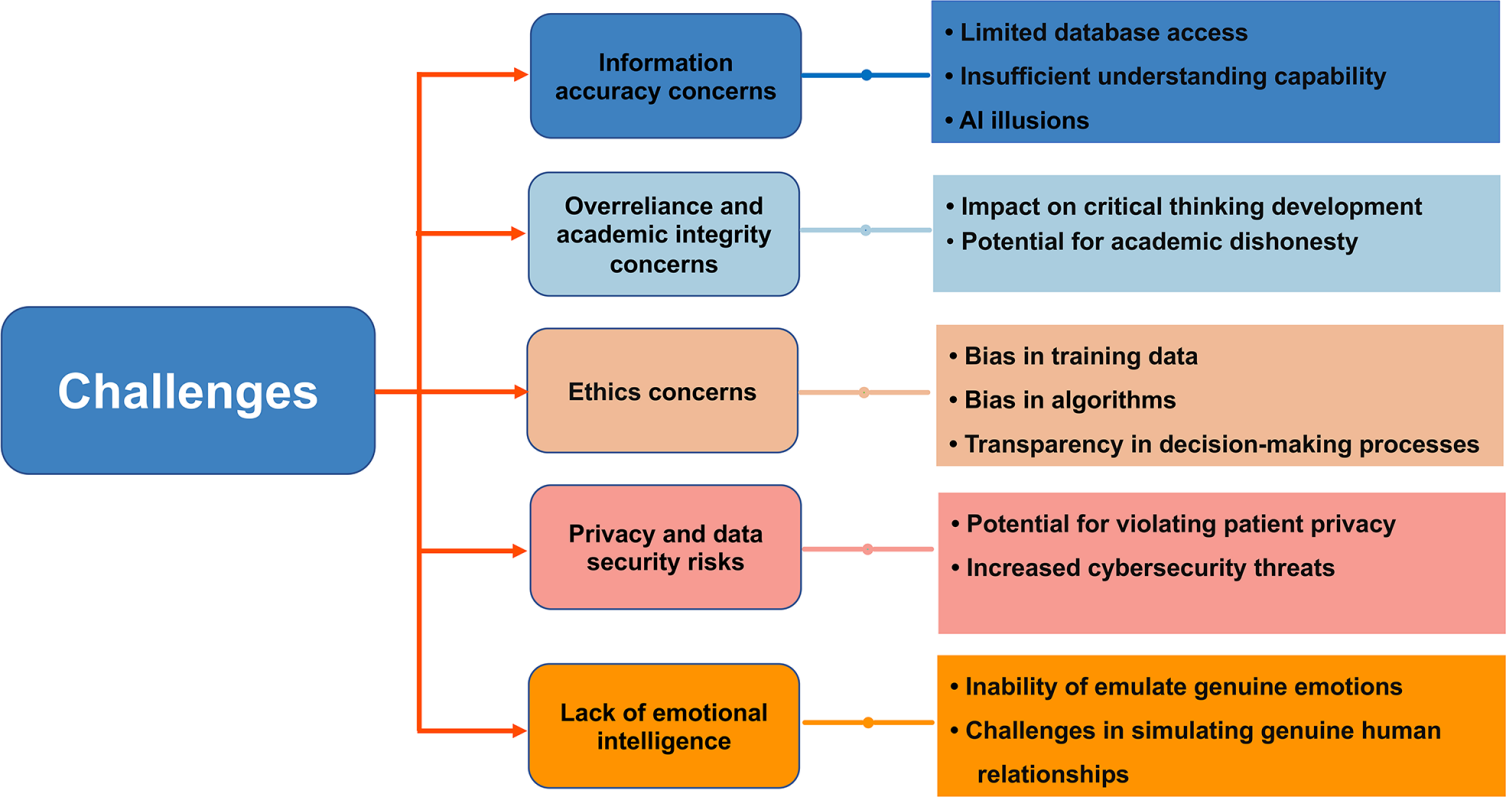
Challenges associated with the use of large language models in medical education. AI: artificial intelligence.

Despite the numerous opportunities in the field of medical education associated with the use of LLMs, this approach also entails a series of challenges and limitations. The challenges posed by LLMs in the context of medical education are summarized in Figure 2.

## Concerns Pertaining to the Accuracy of Information

In medical education, the accuracy of knowledge is of paramount importance, as even minor errors can threaten patient safety. For students who have only limited background knowledge, identifying inaccuracies or misleading information can be particularly challenging. Alkaissi and McFarlane [40] reported that ChatGPT can generate fabricated data, cite nonexistent literature, and provide incorrect citations, a phenomenon known as AI hallucination. Additionally, LLMs may sometimes provide varying responses to identical queries [40,41]. Therefore, the current LLMs cannot yet be fully relied upon in education or research fields. A detailed evaluation of 180 questions answered by the ChatGPT, which was conducted by 33 doctors from 17 medical specialties, revealed that while some responses were highly accurate, significant errors occurred in response to more complex questions [42].Other studies in the medical field have also confirmed this, showing that the ambiguities or errors in LLMs’ responses to complex questions stem from their limited depth of understanding and reasoning capabilities [43,44]. Furthermore, the levels of accuracy exhibited by different language models varied. A study of the accuracy of multiple LLMs in the context of generating answers to dental questions revealed that ChatGPT-4 was significantly more accurate than ChatGPT 3.5, Google Bard, and Microsoft Bing Chat. However, all these LLMs provided irrelevant information, vague answers, or even entirely incorrect responses [38]. Similar conclusions have been reported in several other studies [45-47]. This limitation is primarily due to the training data used for LLMs, which are largely drawn from the internet and often lack rigorous screening and quality control. Consequently, the inclusion of errors, biases, or outdated information directly impacts the accuracy of the outputs of LLMs. Additionally, while current LLMs such as Google Gemini can directly incorporate references from open-access journals into their responses, thus significantly enhancing confidence in the accuracy of the information, access to the subscription sections of certain authoritative databases, such as PubMed, remains limited. This constraint continues to impede the accuracy and comprehensiveness of their responses [48,49].

## Concerns Pertaining to Overreliance and Academic Integrity

Students may depend excessively on LLMs to perform learning tasks, such as writing papers or preparing for examinations. While the content generated by LLMs is original, students who submit text generated by LLMs directly as their own work might engage in plagiarism and academic dishonesty [34,50]. In particular, the integration of LLMs with search engines, which allow direct access to original texts and multimedia, significantly increases the risk of plagiarism. One study indicated that when university professors are presented with abstracts generated by LLMs and those written by humans, they struggle to determine whether these abstracts were authored by a machine or by a human [31]. In a study conducted in 2023, reviewers were able to identify only 63% of fabricated abstracts generated by ChatGPT [27]. This finding suggests that the quality of the text produced by LLMs has equaled or exceeded the quality of the text produced by professionals, thereby providing opportunities for students to engage in academic misconduct by using LLMs to generate summaries, data, or even complete drafts of papers. Furthermore, the ability of LLMs to achieve high scores on exams such as the United States Medical Licensing Exam, as mentioned previously, inevitably raises the possibility of students using them to cheat on exams. Overreliance on LLMs not only entails the risk of academic misconduct but also may impair students’ development of independent research and critical thinking abilities, causing them to have only a shallow grasp of topics and affecting their ability to accurately diagnose complex medical conditions. According to a survey of 370 undergraduate medical students in India, more than 53% of these medical students expressed concerns about the possibility that the widespread application of LLMs might lead to such overreliance, which could hinder their development of clinical reasoning skills [51]. Mechanically extracting answers from LLMs inevitably deprives students of invaluable face-to-face engagement with teachers and peers, thus impeding the development of critical thinking skills, which must be refined through dynamic and diverse intellectual exchanges. Therefore, while LLMs offer significant reference value in medical education, it is crucial to establish appropriate guidelines and balanced strategies to ensure that students can develop independent thinking skills and clinical judgment.

## Bias and Transparency

In medical education, when LLMs are employed, it is essential to scrutinize potential biases in the training data and algorithms underlying these LLMs as well as to address the opacity of the models’ decision-making processes [52]. If an LLM overemphasizes a specific treatment while neglecting others, it might bias students’ perceptions, potentially instilling in them a narrow view of patient care. In January 2024, Zack et al [53] used the Azure OpenAI application interface to evaluate ChatGPT-4 and discovered that the model inadequately represented demographic diversity in terms of medical conditions. It consistently generated clinical vignettes that reinforced stereotypes associated with specific demographic groups. Furthermore, the differential diagnoses produced by the ChatGPT-4 regarding standardized clinical vignettes tended to include stereotypical biases pertaining to certain races, ethnicities, and genders. The assessments and plans generated by the model were significantly linked to demographic attributes; the model thus often recommended more costly procedures and exhibited disparities in terms of patients’ perceptions. This widespread unfair response primarily arises from training data and algorithmic biases. Even if the training data are unbiased, bias in the algorithmic design of LLMs can still lead to biased outputs [54]. The harm of LLMs that output-biased information lies not only in the decline of teaching quality but also in their potential to distort medical students’ ethical principles, leading to unfair treatment of clinical patients and even endangering their health and lives.

When faced with responses from LLMs, users often struggle to understand how these responses are generated or the underlying logic, a phenomenon known as the “black box” effect, indicating a lack of transparency in the model [55]. In medical education, the lack of transparency in LLMs may prevent teachers and students from accurately assessing the accuracy of information, posing risks to patient safety in clinical practice [56]. Additionally, failure to comprehend the logic behind LLMs’ responses may lead to rigid thinking among students, overlooking the importance of logical thinking in medical curriculum learning and thereby weakening the cultivation of critical thinking.

## Risks Pertaining to Privacy and Data Security

AI has long been used to assist in facilitating the diagnosis and treatment of diseases by examining patients’ medical histories and examination images [36]. In medical education, when sensitive patient information such as names, ages, diagnoses, and other details is input for case-based teaching simulations or even as raw data for training LLMs, as well as CT or MR images containing patient information for image interpretation or diagnostic purposes, LLMs may inadvertently disclose patient privacy during the reasoning process or through their “memory” effects [52,57]. Even more concerning, current research has shown that even anonymized information could lead to privacy breaches through cross-referencing with other available web-based data, resulting in the reidentification of personal information [57]. Studies have demonstrated that with only 15 demographic characteristics, 99.98% of personal information can be reidentified [58]. As a cloud-based service, LLMs face various cybersecurity risks regarding data storage and processing, including hacking attacks or the infiltration of malicious software. Any such security vulnerabilities could lead to the leakage of sensitive data, thereby leading to significant risks and legal liabilities [59]. Companies that develop chatbots, such as OpenAI, may employ users’ personal information for various purposes, including service analysis, improvement, and research. Such companies also reserve the right to share users’ personal information with third parties without prior notification or explicit consent from users [60]. All these factors introduce risks pertaining to privacy and data security to the process of medical education.

## Lack of Emotional Intelligence

Cultivating empathy is a cornerstone of medical education. Although language models such as the ChatGPT can be trained to mimic empathetic language and used to facilitate patient communication [61], artificial empathy cannot replicate genuine, subtle empathy on the part of health care providers, which is crucial for patient care and can easily be identified by patients [62]. Prof Marc Succi from Harvard University believes that excessive reliance on artificial emotional communication in medical settings can exacerbate societal loneliness [63]. The subtlety, profundity, and diversity of empathy that is expressed in the context of human-to-human interactions are currently irreplaceable in LLMs. When LLMs simulate patients, all the emotional responses they exhibit are expressed as textual descriptions, thus preventing the student from interpreting or responding to nonverbal cues such as facial expressions, tone of voice, and gestures. These cues are even more crucial for genuine human empathy than verbal expressions [64]. The empathy conveyed through the programmed responses of LLMs poses a challenge for medical students’ attempts to acquire the basic experience necessary to engage in interpersonal relationships by interacting with these models. The establishment and development of empathy also involve addressing the inherent complexity and uncertainty associated with real interactions, in which context the diverse backgrounds, beliefs, and values of real patients lead to a wide range of emotional expressions in interpersonal communication. LLMs struggle to simulate such personalized and diverse emotional responses. Furthermore, cultivating empathy requires ongoing practice over time. LLMs are static models that cannot engage in long-term interaction and provide personalized feedback; accordingly, they are unable to facilitate the deep practices necessary for students to develop empathy.

## Changes in Teaching Methods

**Figure 3.**
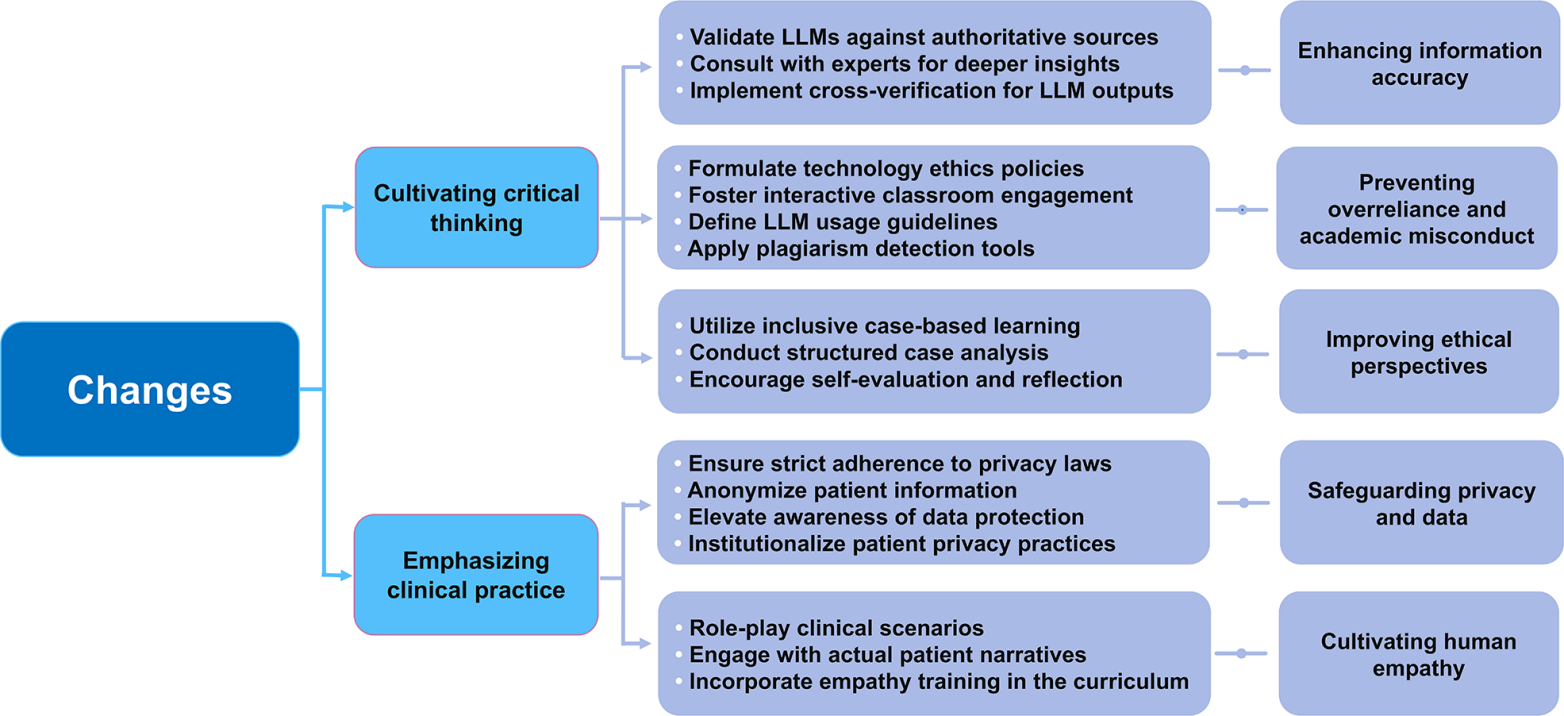
Main content of adaptive changes in educators’ teaching methods. LLM: large language model.

LLMs are currently revolutionizing numerous aspects of medical education. As we exploit the power of these advanced technologies to promote innovation in medical education, we must also address the various challenges they entail. In the context of teaching activities, educators must continue to play a leadership role in the classroom, emphasize the value of traditional classroom settings, enhance students’ critical thinking abilities, and highlight the crucial role of practical experience. In this manner, we can not only benefit from the advantages of LLMs but also ensure the comprehensiveness and effectiveness of the educational process. The specific content of the adaptive changes made by educators in response to the challenges entailed by LLMs is summarized in Figure 3.

To ensure the reliability of the information imparted during medical education, educators must exercise critical scrutiny, especially in light of students’ tendency to trust their teachers to a considerable extent. Compared to one-on-one human-machine interactions, one-to-many scenarios during teaching may exacerbate the adverse effects of misinformation transmission. When using chatbots such as Microsoft’s Copilot or Google’s Gemini, which can access and cite real-time data directly from the internet, it is crucial to recognize that medical students may lack the necessary skills to evaluate the authenticity of such information. Therefore, employing multiple methods to verify information and teaching students how to discern the truthfulness of information has become an urgent and important educational need. Educators should corroborate AI-generated content by providing references to trusted sources such as PubMed, the Cochrane Library, or UpToDate. Additionally, seeking insights from subject matter experts can provide valuable perspectives on complex or nuanced medical topics. Cross-verification, which involves comparing the responses provided by various language models, serves as another means of such validation, which can lead to further investigation of discrepancies. This not only involves effectively transmitting precise knowledge but also helps in developing students’ critical thinking skills.

Simply prohibiting the use of language models to address challenges pertaining to academic integrity is not a practical solution. Educational institutions must establish explicit policies regarding academic misconduct in the context of these technologies. Educators should clearly define how assignments should be completed and inform students of the consequences of academic dishonesty. Just as the response to King’s [34] inquiry to ChatGPT about the strategies college professors should use to prevent students from cheating with ChatGPT suggested, designing assignments that incorporate a variety of assessment methods is an effective approach. This approach includes changing traditional homework methods and supervising students’ independent completion of assignments related to specific topics in class. The introduction of classroom presentations, group discussions, practical activities, and video productions, which are tasks that LLMs cannot complete independently, can highlight students’ knowledge and skills. Additionally, when students inevitably use LLMs to complete assignments, they should be required to clarify the role of LLMs in their work and provide examples of such human-computer interactions for reference. Educators can also use plagiarism detection software to identify copied or unoriginal content in submitted papers [34]. These changes in teaching methods can not only foster critical thinking but also prevent overreliance on AI as well as academic dishonesty.

The development of critical thinking skills is also essential in light of the biases and unfairness entailed by LLMs. Although educators may not have the capacity to alter the biases inherent in training data or LLM algorithms, it is imperative for us to scrutinize the outputs of LLMs critically and to cultivate a similar critical perspective on the part of our students. When case-based instruction is employed, inclusivity should be ensured in terms of diverse populations and medical scenarios to avoid reinforcing stereotypical associations with specific demographic groups or medical conditions. LLM-generated clinical vignettes should be used as pedagogical tools by prompting students to engage in structured activities such as group discussions, role-playing, or debates. These activities should focus on dissecting the reasoning process, diagnostic conclusions, and therapeutic recommendations presented in the cases in question, thus emphasizing the process of identifying and critiquing potential biases and logical inconsistencies. Additionally, the broader ethical, societal, and cultural ramifications of the deployment of LLM-generated insights in medical decision-making should be explored. Students should be encouraged to engage in reflective practices after interacting with LLMs, thus prompting them to assess their decision-making trajectory and outcomes introspectively and to recognize and address their inherent biases and cognitive patterns.

Educators should prioritize hands-on experience and advocate for experiential learning anchored in authentic clinical settings. Regarding data privacy and security, it is crucial to adhere strictly to legal standards regarding patient confidentiality. Educators should aim to minimize the use of actual patient data in LLMs and prefer to use synthetic or hypothetical scenarios to avert any potential privacy breaches. In cases in which it is necessary to input patient-specific medical history, examination images, and other sensitive information into LLMs, it is imperative for students to obtain informed consent from the patient, obtain the necessary approval from an ethics committee, and implement appropriate data anonymization measures. Additionally, it is vital to provide medical students with comprehensive education and training regarding patient privacy protection, including lawful and ethical procedures for the collection, use, and storage of patient information. By actively engaging in clinical practices that emphasize the importance of patient privacy and preventing data leakages, medical students can cultivate a profound understanding of the legal and ethical responsibilities associated with patient privacy protection.

An emphasis on clinical practice is essential for the development of fundamental clinical skills and the promotion of profound empathic connections, both of which are rooted in medical practice. To cultivate empathy, practical teaching methods such as role-playing and simulated patient interactions can be used to enhance students’ effective doctor-patient communication skills. These skills include listening techniques, nonverbal communication, and appropriate expressions of empathy and care. Engaging with real patients allows medical students to observe, learn from, and emulate the empathetic behaviors of experienced instructors and to apply these insights to patient care. Accumulating experiences in empathy by engaging in diverse patient interactions and a process of immersive reflection can enable students to understand and empathize with patients deeply, thereby equipping them to address the complexities of patient care effectively. Moreover, the incorporation of disciplines such as psychology and other disciplines in the humanities into medical curricula can provide students with a theoretical foundation for understanding patients’ emotional and psychological states, thereby enriching the practical application of their skills in clinical settings.

We must acknowledge the positive role played by LLMs in education, and educators must ensure that this technology can complement traditional teaching methods rather than replace them. Teachers should respond to the opportunities and challenges associated with the use of LLMs by adapting their teaching methods and content, promoting critical thinking among students, and emphasizing the importance of practical experience to ensure that teachers continue to play a leading role in medical education. In the future, establishing unified ethical principles or guidelines for the application of LLMs in medical education could better guide educators and students on how to effectively and safely use LLMs. Such a balanced approach can ensure that future medical professionals are better equipped by combining technological advancements with the enduring values of medical education.

## Conclusion

We explored the diverse opportunities and challenges associated with the use of LLMs in medical education. While LLMs offer new avenues for exploration and innovation in the context of medical education, educators must recognize that these technologies serve as supplementary tools to traditional teaching methodologies. The expertise and foresight of educators remain paramount regarding safeguarding the quality of teaching. Educators must possess the ability to exploit these emerging technologies while continuing to play a guiding role in student learning, nurture critical thinking skills, and consistently emphasize the significance of clinical practice in medical education, thus ensuring that students use LLMs judiciously and efficaciously. By obtaining a comprehensive understanding of the strengths and limitations of LLMs and making corresponding adjustments to their teaching approaches, educators can steer medical education toward a more innovative, intelligent, and practice-oriented future.

## Abbreviations

AI: artificial intelligence
CT: computed tomography
LLMs: large language models
MRI: magnetic resonance imaging
SPs: standardized patients
